# Prognostic and Predictive Value of a Long Non-coding RNA Signature in Glioma: A lncRNA Expression Analysis

**DOI:** 10.3389/fonc.2020.01057

**Published:** 2020-07-24

**Authors:** Yuan-Bo Pan, Yiming Zhu, Qing-Wei Zhang, Chi-Hao Zhang, Anwen Shao, Jianmin Zhang

**Affiliations:** ^1^Department of Neurosurgery, Second Affiliated Hospital, School of Medicine, Zhejiang University, Hangzhou, China; ^2^Department of General Surgery, Shanghai Ninth People's Hospital, Shanghai Jiaotong University School of Medicine, Shanghai, China; ^3^Division of Gastroenterology and Hepatology, Key Laboratory of Gastroenterology and Hepatology, Ministry of Health, Renji Hospital, School of Medicine, Shanghai Jiao Tong University, Shanghai, China; ^4^Shanghai Institute of Digestive Disease, Shanghai Jiao Tong University, Shanghai, China; ^5^Brain Research Institute, Zhejiang University, Hangzhou, China; ^6^Collaborative Innovation Center for Brain Science, Zhejiang University, Hangzhou, China

**Keywords:** glioma, lncRNA, prognosis, survival, chemotherapy, nomogram

## Abstract

The current histologically based grading system for glioma does not accurately predict which patients will have better outcomes or benefit from adjuvant chemotherapy. We proposed that combining the expression profiles of multiple long non-coding RNAs (lncRNAs) into a single model could improve prediction accuracy. We included 1,094 glioma patients from three different datasets. Using the least absolute shrinkage and selection operator (LASSO) Cox regression model, we built a multiple-lncRNA-based classifier on the basis of a training set. The predictive and prognostic accuracy of the classifier was validated using an internal test set and two external independent sets. Using this classifier, we classified patients in the training set into high- or low-risk groups with significantly different overall survival (OS, HR = 8.42, 95% CI = 4.99–14.2, *p* < 0.0001). The prognostic power of the classifier was then assessed in the other sets. The classifier was an independent prognostic factor and had better prognostic value than clinicopathological risk factors. The patients in the high-risk group were found to have a favorable response to adjuvant chemotherapy (HR = 0.4, 95% CI = 0.25–0.64, *p* < 0.0001). We built a nomogram that integrated the 10-lncRNA-based classifier and four clinicopathological risk factors to predict 3 and 5 year OS. Gene set variation analysis (GSVA) showed that pathways related to tumorigenesis, undifferentiated cancer, and epithelial–mesenchymal transition were enriched in the high-risk groups. Our classifier built on 10-lncRNAs is a reliable prognostic and predictive tool for OS in glioma patients and could predict which patients would benefit from adjuvant chemotherapy.

## Introduction

About 81% of malignant brain tumors are due to gliomas (1). Maximal surgical resection followed by adjuvant chemotherapy or radiotherapy is the standard treatment for glioma patients (2). Based on histopathological features, glioma can be divided into four groups (Grades I, II, III, and IV) (3).

The histopathological grading system is the key determinant for prognostic prediction and risk stratification for treatment decisions. Although patients with the same grade of glioma receive similar treatment, their clinical prognoses vary widely (4, 5). Therefore, the current histologically based grading system is not sufficient to predict which patients will have better outcomes or benefit from adjuvant chemotherapy. Prognostic differences may be attributed to biological heterogeneity. Molecular investigation could provide biomarkers for predicting OS and the benefits from adjuvant chemotherapy and for guiding treatment decisions for patients in different risk groups. Thus, it is necessary to augment the prognostic and predictive value of the histologically based grading system, which could be achieved with the use of new biomarkers.

Long non-coding RNAs (lncRNAs) belong to the family of non-coding RNAs and range from 200 nucleotides to multiple kilobases in length (6). Accumulating evidence suggests that dysregulation of lncRNAs has been associated with glioma (7–9), and some of them have been implicated in prognostication and diagnosis (9, 10). Integrating multiple biomarkers rather than a single one into a single model could improve the prognostic value substantially (11, 12). When over tens of thousands of biomarkers are simultaneously detected using microarrays or RNA-seq high-throughput techniques, the number of covariates is close to, or greater than, the number of observations. The Cox proportional hazards regression analysis, being the most universal method for evaluating prognosis and survival, is not suitable for high-dimensional data where the ratio of samples to variables is very small (13, 14). Instead, LASSO can eliminate this limitation and has been widely adopted for optimal selection of prognostic genes (15–18).

In our study, 1,094 glioma samples from different populations were analyzed, and a multi-lncRNA-based classifier was established using a LASSO Cox regression model to predict OS and benefit from adjuvant chemotherapy for patients with glioma who had already had surgery. We assessed the prognostic and predictive accuracy of the classifier in the training set and validated it in one internal testing group and two independent patient groups. We also compared its prognostic and predictive efficacy to those of every single lncRNA and clinicopathological risk factor. Furthermore, we assessed the prognostic value of the classifier when stratified by these clinicopathological risk factors. In addition, we combined the multi-lncRNA-based classifier and clinical variables to build, for the first time, a nomogram for glioma, which achieves excellent prediction compared to clinicopathological risk factors for OS.

## Materials and Methods

### Dataset Preparation

In total, 1,094 gliomas derived from three independent datasets were studied here, comprising one dataset from the Chinese Glioma Genome Atlas (CGGA), one dataset from The Cancer Genome Atlas (TCGA), and one dataset (GSE16011) from the Gene Expression Omnibus (GEO). The samples without clinical survival information in these datasets were filtered out. The dataset from GEO (GSE16011) was processed using the chip platform (Affymetrix Human Genome U133 Plus 2.0 Array, Santa Clara, CA, USA), which has been most commonly applied in transcriptome analysis.

### Data Processing and lncRNA Profile Mining

For microarray data (GSE16011), the raw probe-level data in each CEL file was processed via the robust multi-array average (RMA) algorithm of the Affy package (19). For genes that match multiple probes, we used average probe values for expression value (20). For TCGA data, the count files downloaded from the data portal of TCGA were normalized through the edgER package (21). For the CGGA dataset, RNA-seq data and corresponding clinical information were downloaded from the data portal of CGGA. Missing data in these gene expression matrixes were assigned using the k-Nearest Neighbor (KNN) approach (k = 10) (22). lncRNA profile mining was achieved by the established mining approach (23). Firstly, we converted the probe ID or transcript ID or Ensembl gene ID to gene name. Secondly, gene names were mapped to an annotation file of GENCODE v30, and long non-coding RNAs were extracted. Finally, we retained the 361 lncRNAs that were annotated in all three datasets (CGGA, TCGA, and GSE16011) to ensure the validation of the lncRNA-based classifier. The lncRNA expression data was Z-score transformed to avoid systematic error across different experiments.

### Development and Validation of the 10-lncRNA-Based Classifier

The relationship between lncRNA profile and patient OS was evaluated with univariable Cox regression analysis using R software. Due to the high dimensionality of the gene expression data, there could be overfitting during analysis. LASSO is the standard high-dimensional data analysis approach, as it has the potential to increases prediction precision as well as interpretation (15, 24). Consequently, we applied this model to choose the most suitable prognostic indicators and to build the lncRNA classifier. R software version 3.4.3 and the “glmnet” package (R Foundation for Statistical Computing, Vienna, Austria) were applied to perform the LASSO Cox regression model study. We created a risk score formula based on the expression levels of the 10 lncRNAs for OS prediction, where risk score = (0.353815^*^expression level of LINC00645) + (0.800399^*^expression level of LINC00339) + (0.681679^*^expression level of ZNF790-AS1) + (0.211079^*^expression level of HOXD-AS2) + (0.120801^*^expression level of RHPN1-AS1) + (0.140439^*^expression level of FOXD2-AS1) – (1.778034^*^expression level of TMEM72-AS1) – (0.404113^*^expression level of ARHGEF26-AS1) – (0.708967^*^expression level of HAR1A) – (0.413111^*^expression level of EPB41L4A-AS1). By design, the X-tile program chooses the optimum cut-off point depending on the uppermost χ^2^ (minimum *p*) value determined via Kaplan–Meier survival analysis and log-rank test (25). The optimum cut-off values in this study were selected via X-tile plots. Using the cut-off values, each dataset was categorized into low- and high-risk groups. The resultant plots were generated by 3.6.1 version X-tile software (School of Medicine, Yale University, New Haven, CT, USA). We explored prognosis or predictive accuracy per variable as well as the 10-lncRNA-constructed classifier with time-dependent ROC analysis (26). R software version 3.4.3 and the “survival” package together with the “riskRegression” package were used to perform time-dependent ROC analysis.

### Statistical Analysis

We compared two groups using the *t-*test for continuous variables and the χ^2^ test for categorical variables. During survival analysis, the Kaplan-Meier process was used to analyze the association between variables and OS and the log-rank test was used for comparison of survival curves. Furthermore, a Cox regression model was applied in standard univariate and multivariate analysis, and Cox regression coefficients were used to generate the nomogram. ROC analysis depending on time was performed to evaluate the nomogram predictive accuracy. 3.4.3 version R software plus the “rms” package were employed in generating nomogram and calibration plots. Decision curve analysis (DCA) was applied to determine the clinical feasibility of the nomogram (27, 28). The most significantly differentially expressed gene sets (*p*-value < 0.001) were selected. The entire statistical analysis was completed using R software version 3.4.3 at a statistical significance of 0.05.

## Results

### The 10-lncRNA-Based Classifier and Patient Survival in the Training Set

**Figure 1:**
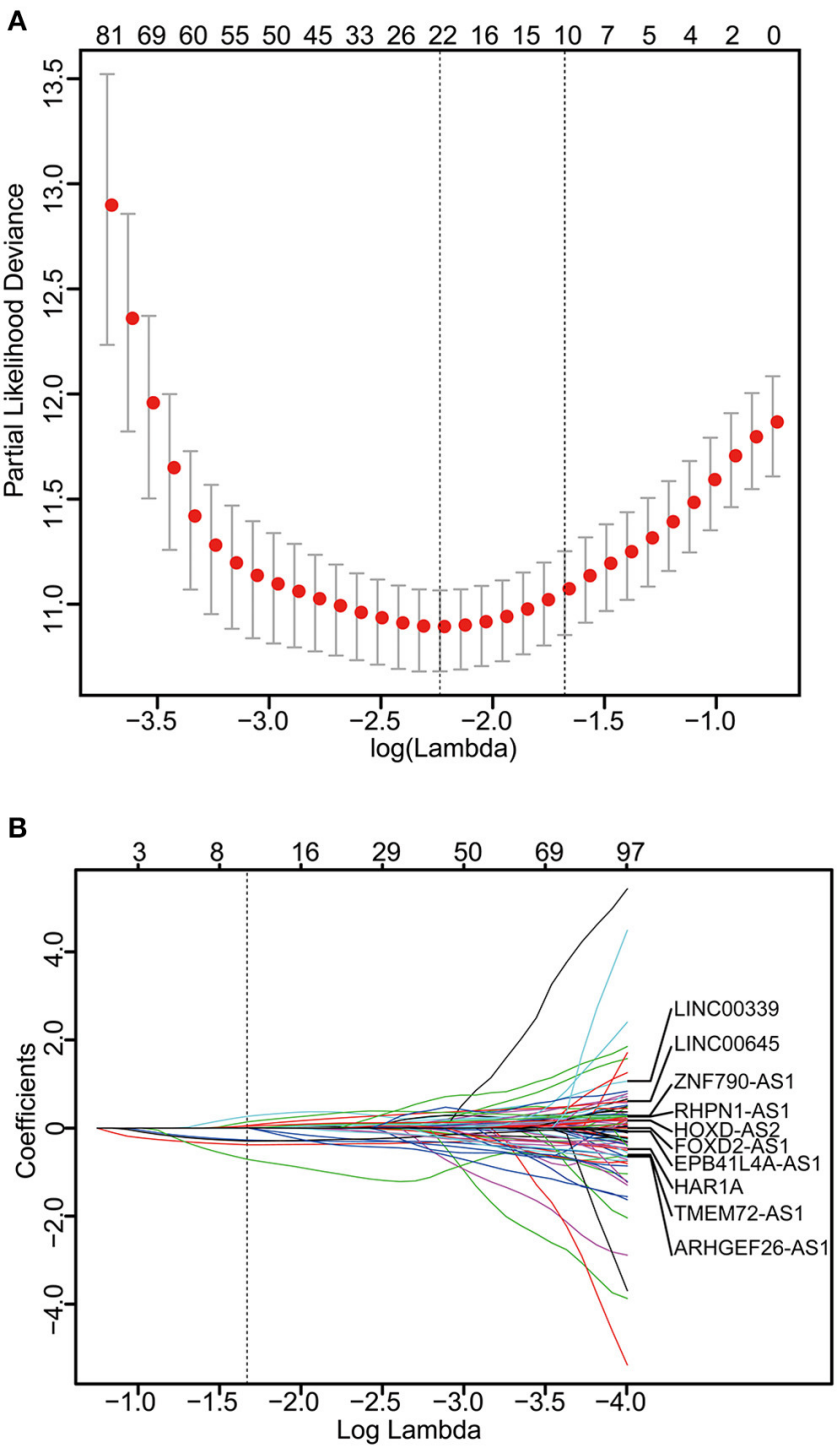
Construction of the 10-lncRNA-based classifier. **(A)** Ten-time cross-validation for tuning parameter selection in the LASSO model. The solid vertical lines are partial likelihood deviance ± standard error (SE). The dotted vertical lines are drawn at the optimal values by minimum criteria and 1-SE criteria. A λ value of 0.187 with log(λ) = −1.678 was chosen by 10-time cross-validation via 1-SE criteria. **(B)** LASSO coefficient profiles of the 176 glioma-prognosis-associated lncRNAs. A dotted vertical line is drawn at the value identified by 10-fold cross-validation.

The 249 glioma samples of the CGGA dataset were randomly assigned to a training set (*n* = 166) and an internal testing set (*n* = 83). The training set was used to detect the prognostic lncRNAs. After using univariable Cox regression to analyze the lncRNA expression data of the training set, we identified 176 lncRNAs whose parameter *p*-value was lower than 0.01. Then, we build a prognostic classifier via the LASSO Cox regression model. A dotted vertical line was drawn (Figures 1A,B) at the value chosen by 10-fold cross-validation. The optimal λ value, 0.187, with log(λ) = −1.678 results in 10 non-zero coefficients. Thus, the 10-lncRNAs were selected from 176 lncRNAs: HAR1A, EPB41L4A-AS1, LINC00339, RHPN1-AS1, FOXD2-AS1, TMEM72-AS1, ARHGEF26-AS1, LINC00645, ZNF790-AS1, and HOXD-AS2 (Figures 1A,B, Table S1). The expression of the 10 lncRNAs had low correlations (Figure S1A). We created a risk score formula based on the expression levels of the 10 lncRNAs for OS prediction (shown in the methods section). Then, we calculated the 10-lncRNA-based risk score for each patient in the training set and ranked them according to their risk scores. Patients in the training set were classified into the high-risk or low-risk group with an optimum cut-off value of 2.11 by using X-tile plots (Figures S1B–S1D). The clinical characteristics of these datasets are listed in Table S2.

**Figure 2:**
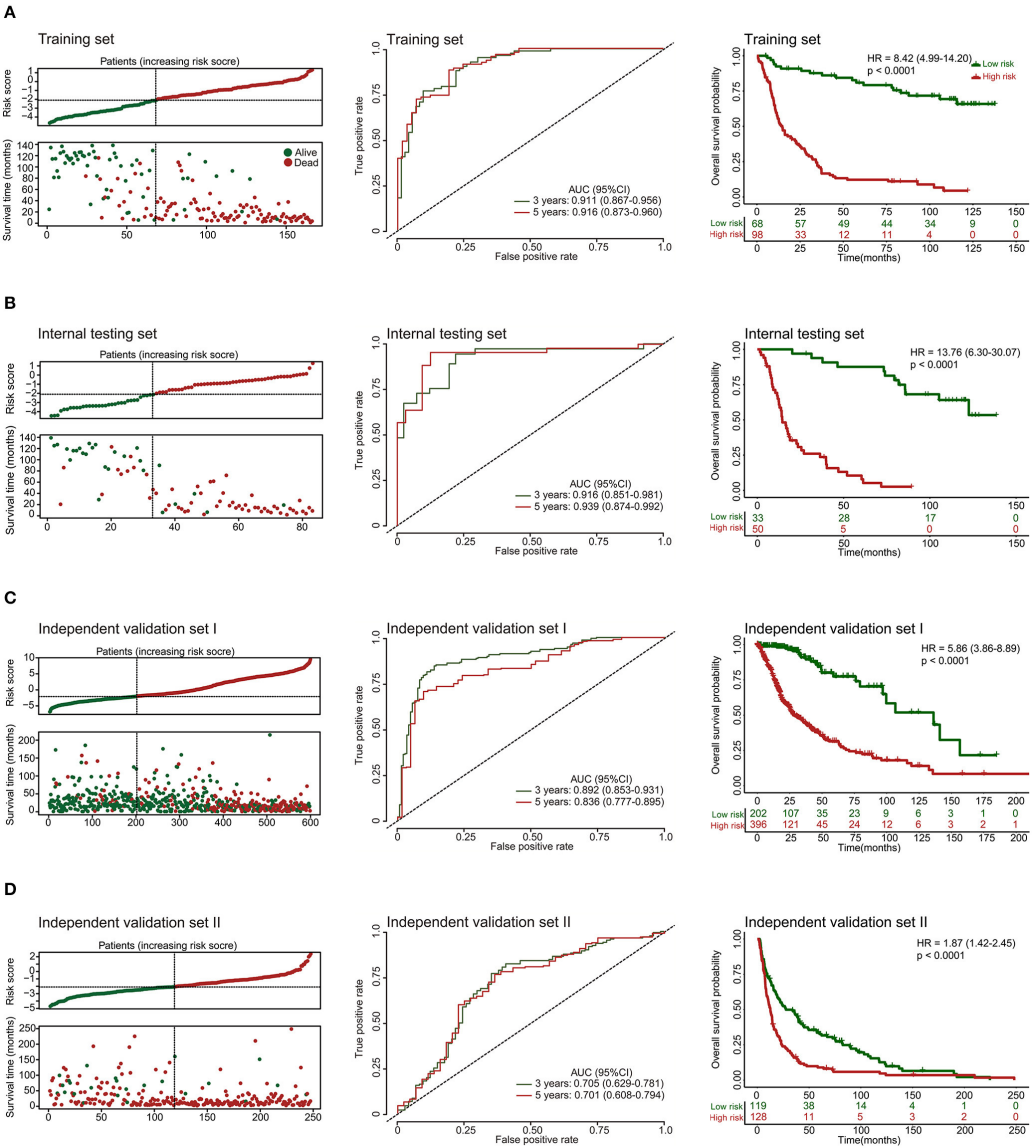
Risk score by the 10-lncRNA-based classifier, patient's survival status and time, time-dependent ROC curves, and Kaplan-Meier survival in the training, internal testing, and two independent validation sets. **(A)** Training set. **(B)** Internal testing set. **(C)** Independent validation set I. **(D)** Independent validation set II. We used AUCs at 3 and 5 years to assess prognostic accuracy and calculated *p*-values using the log-rank test. Data are AUC (95% CI) or hazard ratio (95% CI).

**Figure 3:**
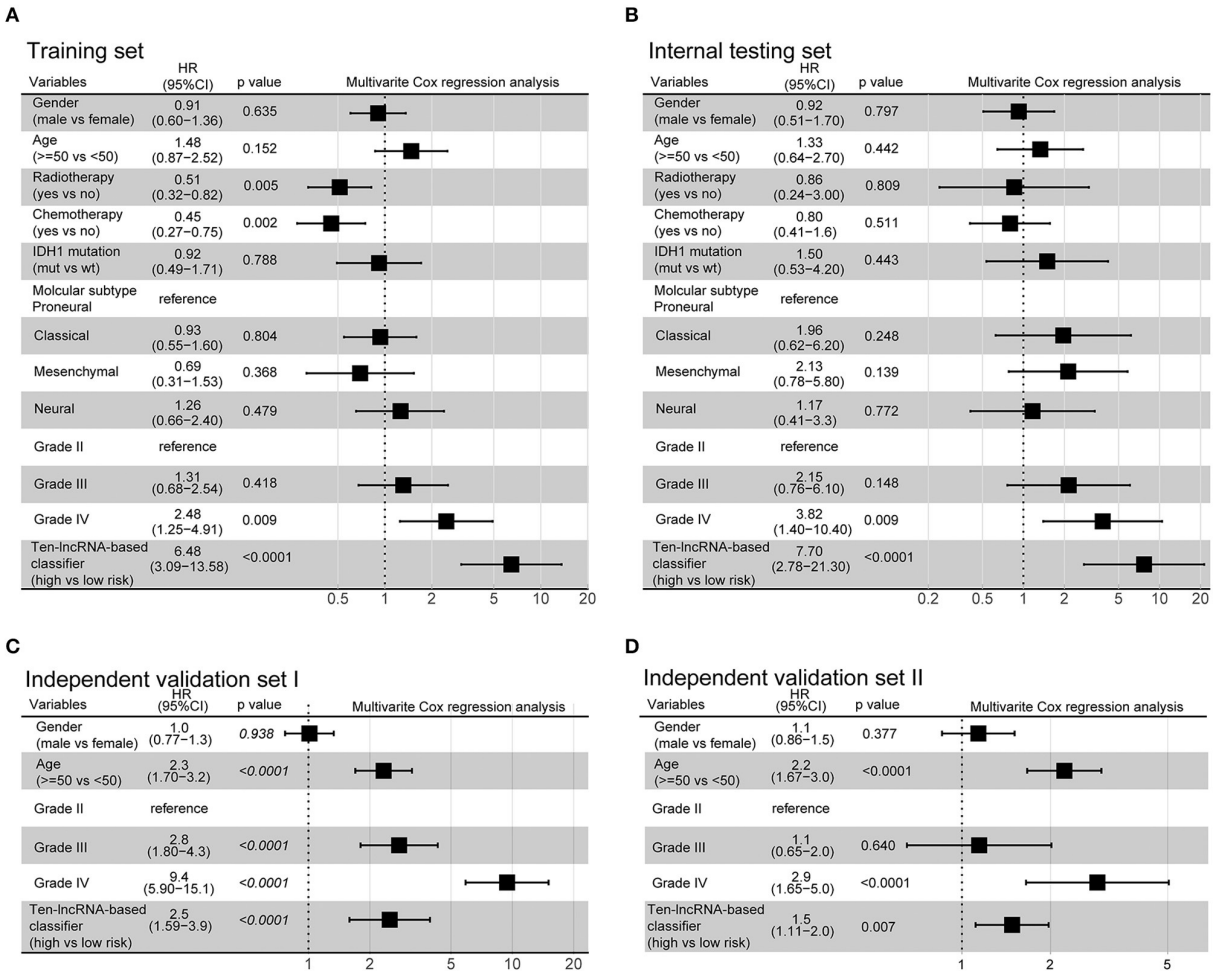
Multivariate analysis based on the 10-lncRNA-based classifier and clinical risk factors in the training, internal testing, and two independent validation sets. **(A)** Training set. **(B)** Internal testing set. **(C)** Independent validation set I. **(D)** Independent validation set II. Solid and black squares represent the HR of death. Close-ended horizontal lines represent 95% CI. We calculated *p*-values using Cox regression hazard analysis.

Additionally, we evaluated the prognostic precision of the 10-lncRNA-based risk score with time-dependent ROC analysis at different follow-up periods (Figure 2A). The Kaplan-Meier survival analysis of the training set revealed that patients in the high-risk group had significantly worse outcomes than those in the low-risk group (Figure 2A). Then, we assessed the distributions of risk scores, survival time, and status and found that patient with lower risk scores generally had better survival than those with higher risk scores (Figure 2A). In addition, the 10-lncRNA-based classifier was a strong variable correlated with prognosis in the univariate Cox regression model (Figure S2A). After multivariable adjustment by clinicopathological factor, the 10-lncRNA-based classifier remained a powerful and independent factor in the training set (Figure 3A).

### Validation of the 10-lncRNA Based Classifier for Survival Prediction in the Internal Testing Set and the Entire CGGA Dataset

To confirm the robustness of the classifier, we validated the 10-lncRNA-based classifier in the internal testing set and the entire CGGA dataset. Using the same risk score formula, we calculated the risk score for each patient and classified them into high-risk and low-risk groups using the same cut-off point (2.11). Consistent with the above finding, the high-risk patients set displayed considerably shorter median OS than the low-risk patients (Figure 2B, Figure S3A). We performed time-dependent ROC analysis to assess the prognostic accuracy of the 10-lncRNA-based risk score at two follow-up times (3 and 5 years) in both sets (Figure 2B, Figure S3A). We also found that patients with a lower risk score mostly had better survival than those with higher risk scores (Figure 2B, Figure S3A). In addition, univariate Cox regression analysis also revealed that the 10-lncRNA-based classifier was a strong factor correlated with prognosis (Figures S2B, S3B). Likewise, after multivariable adjustment by clinicopathological factor, the 10-lncRNA-based classifier remained a powerful and independent factor in the two sets (Figure 3B, Figure S3C).

### Further Validation of the 10-lncRNA-Based Classifier in Two Other Independent Sets

To confirm that the classifier had similar prognostic value in diverse populations, we further used it in two other independent glioma datasets obtained from TCGA (Independent validation set I, *n* = 598) and GSE16011 (Independent validation set II, *n* = 247). The clinical characteristics of the two datasets are listed in Table S2. The two independent sets of individuals were grouped into high-risk and low-risk groups based on their risk score, using the same cut-off point as previously (2.11). Consistent with the findings described above, the 10-lncRNA-based classifier could effectively predict patient OS (Figures 2C,D). Furthermore, univariate Cox regression analysis showed that the 10-lncRNA-based classifier was significantly associated with OS in the two independent sets (Figures S2C, S2D). Moreover, for the multivariable Cox regression model, the 10-lncRNA-based classifier remained an independent variable in the two independent sets (Figures 3C,D).

### 10-lncRNA-Based Classifier Kaplan-Meier Survival Analysis for Gliomas Stratified by Clinicopathological Risk Factors

**Figure 4:**
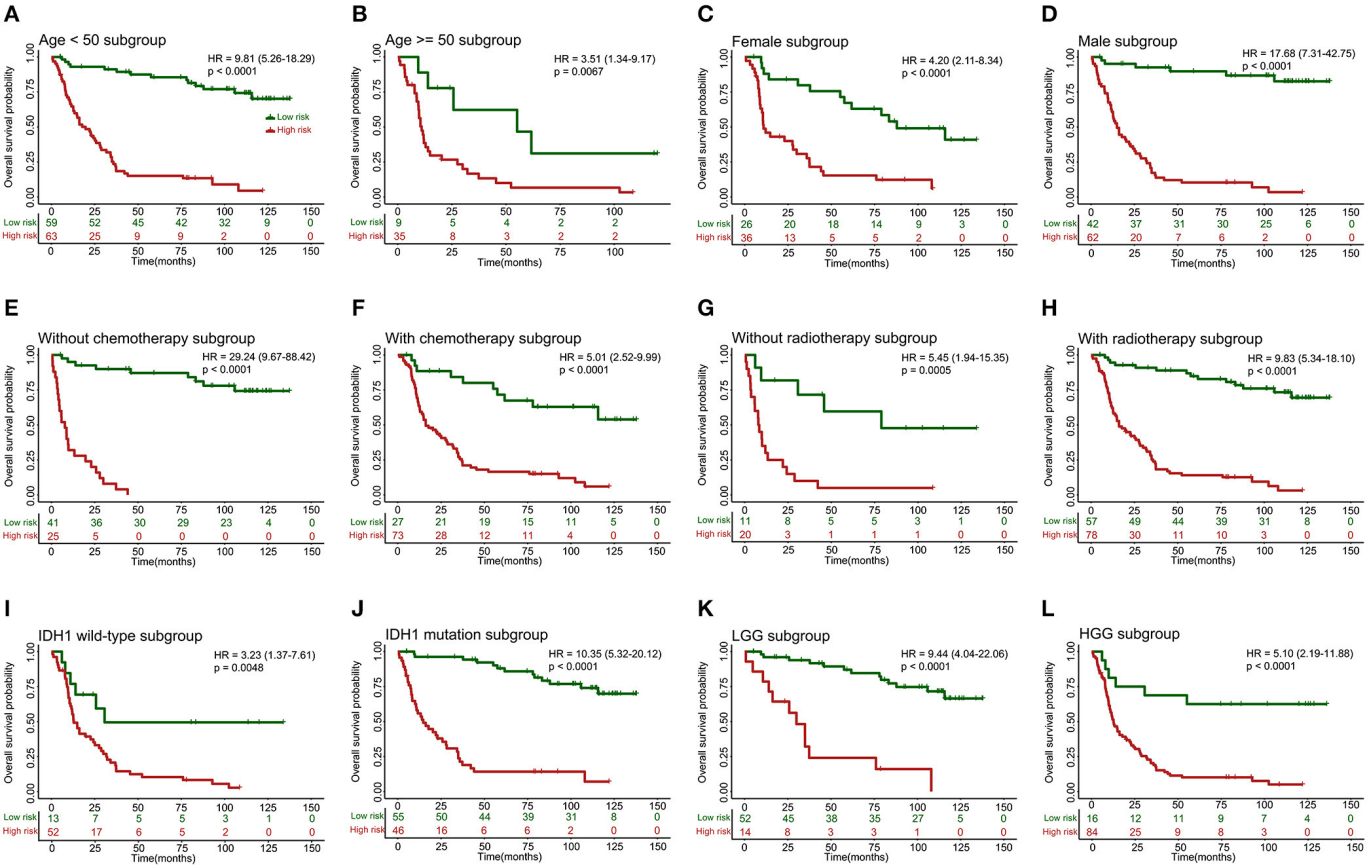
Kaplan-Meier survival analysis for patients in the training set according to the 10-lncRNA-based classifier stratified by clinicopathological risk factors. **(A,B)** Age. **(C,D)** Gender. **(E,F)** Adjuvant chemotherapy. **(G,H)** Radiotherapy. **(I,J)** IDH1 status. **(K,L)** Low-grade glioma (LGG) or high-grade glioma (HGG). We calculated *p*-values using the log-rank test.

When stratified by clinicopathological risk factors, the 10-lncRNA-based classifier remained a clinically and statistically significant prognostic model (Figure 4). For example, the stratification analysis showed that the 10-lncRNA-based classifier could identify patients with different prognoses despite their having the same chemotherapy stratum. In the training set, the 10-lncRNA-based classifier could subdivide the patients with adjuvant chemotherapy (*n* = 100) into those likely to have longer vs. shorter survival (Figure 4F). Similarly, among the patients without adjuvant chemotherapy (*n* = 66), the 10-lncRNA-based classifier could still subdivide them into two subgroups with significantly disparate survival (Figure 4E). In addition, when stratified by other clinicopathological risk factors, including age (age >= 50 vs. age <50), gender, radiotherapy, IDH1 mutation (mutation vs. wild-type), and grade, the classifier remained a strong prognostic tool (Figure 4).

These findings were validated in other datasets (Internal testing set, Figure S4A; Independent validation set I, Figure S4B; Independent validation set II, Figure S4C), which also showed the 10-lncRNA classifier remained an efficient prognostic tool when stratified by clinicopathological risk factors.

### Comparison of Prognostic Accuracy Between the 10-lncRNA-Based Classifier and Any Clinicopathological Risk Factor or Single lncRNA

**Figure 5:**
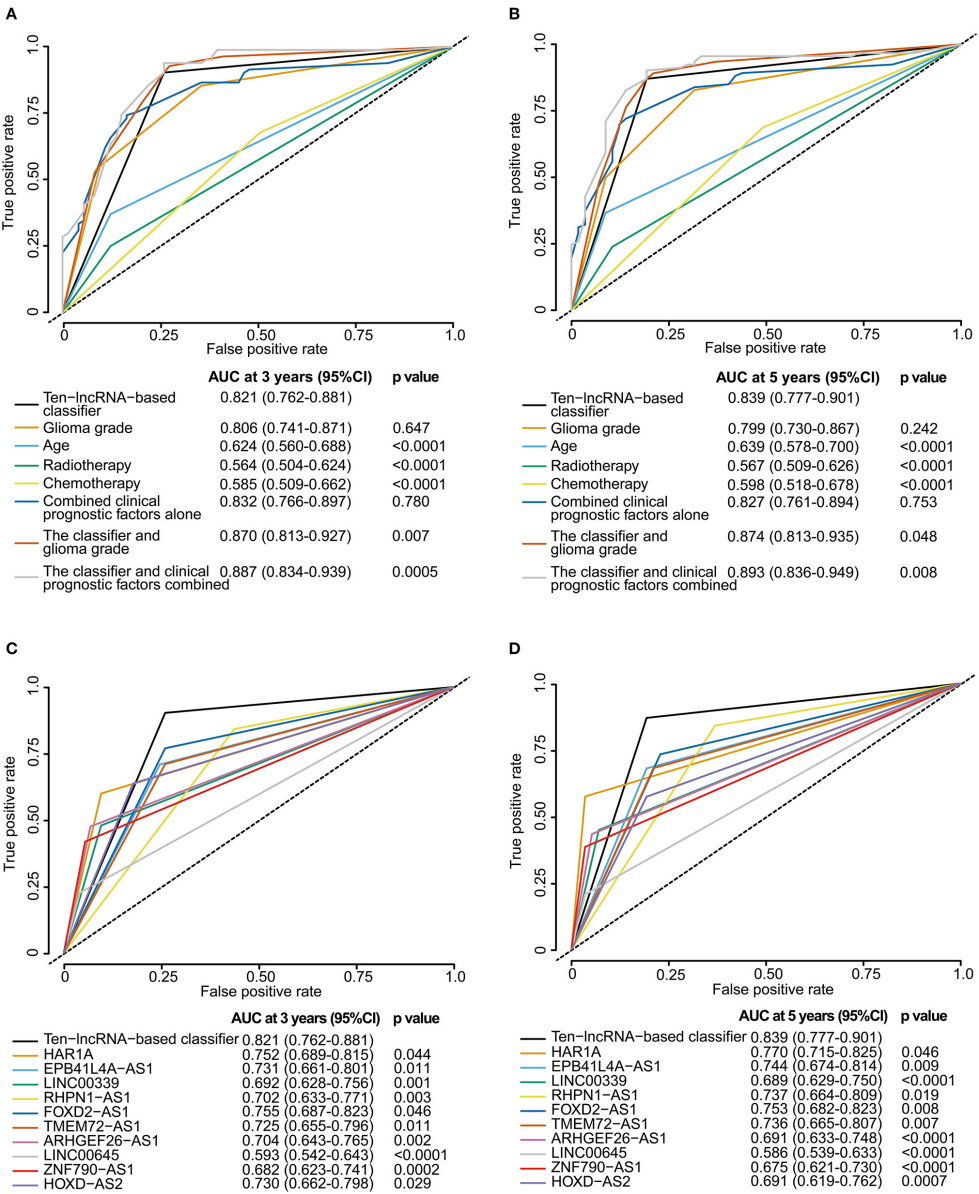
Time-dependent ROC curves comparing the prognostic accuracy of the 10-lncRNA-based classifier with those of clinicopathological risk factors and single lncRNAs in the training set. **(A,B)** Comparisons of prognostic accuracy by the 10-lncRNA-based classifier (high risk vs. low risk), glioma grade, age (age >= 50 vs. age <50), radiotherapy (with vs. without radiotherapy), chemotherapy (with vs. without chemotherapy), combined clinical prognostic factors alone, the classifier and glioma grade combined, or the classifier and clinical prognostic factors combined. **(C,D)** Comparisons of the prognostic accuracy by the 10-lncRNA-based classifier (high risk vs. low risk), HAR1A (high vs. low expression), EPB41L4A-AS1 (high vs. low expression), LINC00339 (high vs. low expression), RHPN1-AS1 (high vs. low expression), FOXD2-AS1 (high vs. low expression), TMEM72-AS1 (high vs. low expression), ARHGEF26-AS1 (high vs. low expression), LINC00645 (high vs. low expression), ZNF790-AS1 (high vs. low expression), or HOXD-AS2 (high vs. low expression). The optimum cut-off points for the 10-lncRNAs were generated using X-tile plots. **(A,C)** AUC at 3 years. **(B,D)** AUC at 5 years. The *p*-value shows the AUC at 3 or 5 years for the 10-lncRNA-based classifier vs. the AUC at 3 or 5 years for other features.

We perform time-dependent ROC analysis for the training set to compare the prognostic precision of the classifier with that of any clinicopathological risk factor or single lncRNA. The area under the curve (AUC) was assessed and compared among these factors. As shown in Figure 5A, ROC time-based analysis at 3 year follow-up revealed that the 10-lncRNA-based classifier showed significantly higher prognostic accuracy than age, radiotherapy, or chemotherapy. There was no significant difference between the AUC of the 10-lncRNA based classifier and that of glioma grade (Figure 5A). However, the AUC of the 10-lncRNA based classifier combined with glioma grade was significantly greater than that of the 10-lncRNA based classifier alone (Figure 5A). Furthermore, the AUC of the 10-lncRNA based classifier combined with all clinicopathological risk factors (glioma grade, age, radiotherapy, and chemotherapy) was also significantly greater than that of the 10-lncRNA based classifier alone (Figure 5A). Time-dependent ROC analysis showed similar results for 5 year follow-up (Figure 5B).

X-tile plots were used to produce the best cut-off points for the 10-lncRNAs in the training set (Figure S5). According to the cut-off point of each lncRNA, patients were divided into the high or low expression group. Time-dependent ROC analysis at 3 year follow-up showed that the 10-lncRNA-based classifier had significantly higher prognostic accuracy than any single lncRNA alone (Figure 5C). Time-dependent ROC analysis showed similar results for 5 year follow-up (Figure 5D).

Although the prognostic ability of the 10-lncRNA-based classifier was equivalent to that of glioma grade, the 10-lncRNA classifier could add prognostic value to clinicopathological prognostic features, which indicated that the 10-lncRNA-based classifier combined with glioma grade or all clinicopathological risk factors could have a stronger power for OS prediction in time-dependent ROC analysis at 3 or 5 year follow-up.

### The 10-lncRNA-Based Classifier Can Identify Glioma Patients Suitable for Adjuvant Chemotherapy

**Figure 6:**
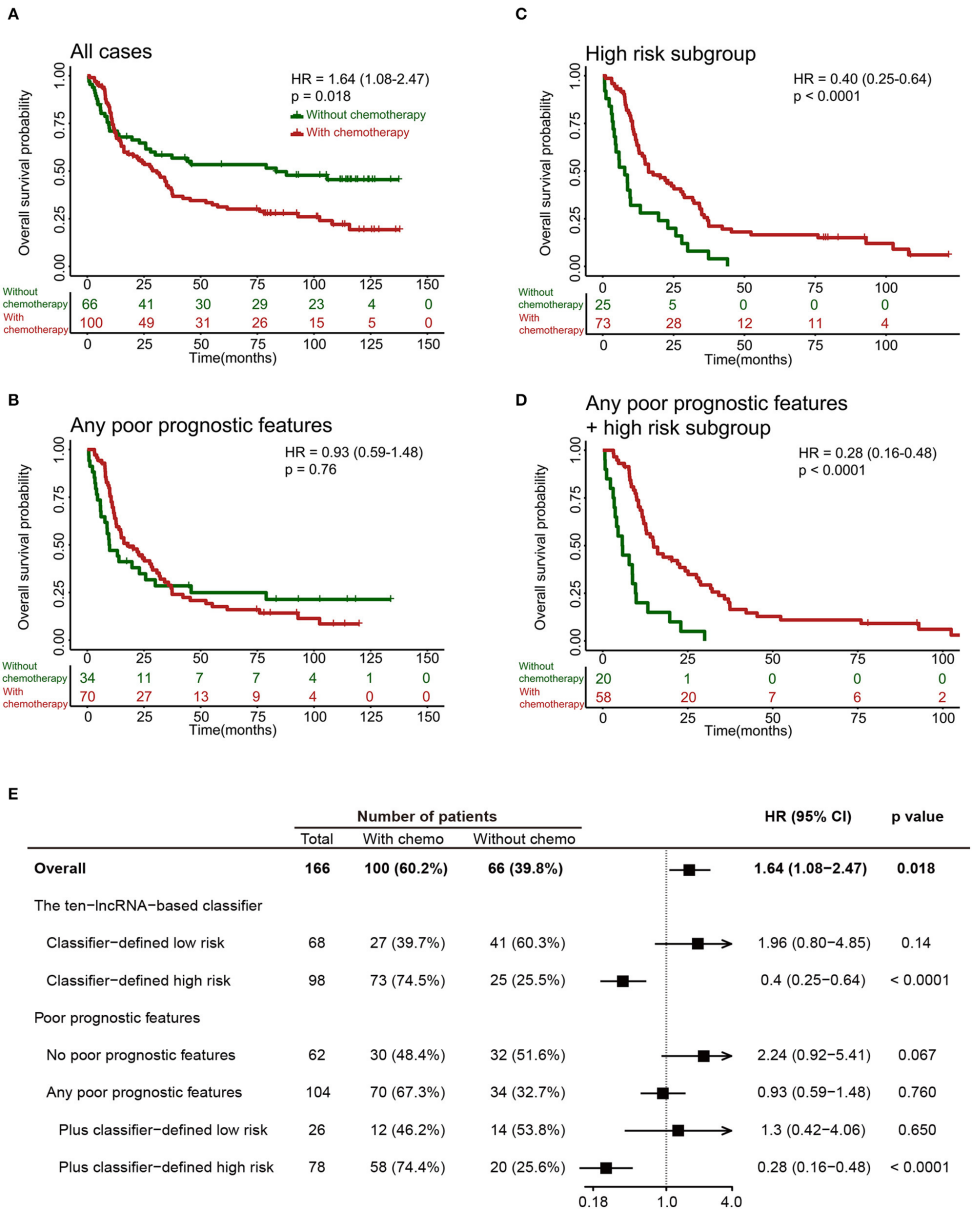
Effect of adjuvant chemotherapy in different subgroups. **(A–D)** Kaplan-Meier survival analysis for patients in different subgroups, which were stratified by the receipt of chemotherapy. **(E)** Effect of adjuvant chemotherapy on OS in different subgroups.

It was noteworthy that adjuvant chemotherapy did not enhance survival in all 249 patients in the training set (Figures 6A,E) or in patients with any poor prognostic features (Age >=50, grade IV, without radiotherapy or IDH1 wild-type; Figures 6B,E). Results from an *ad-hoc* exploratory subgroup analysis using our 10-lncRNA-based classifier revealed that patients in the classifier-defined high-risk group had a favorable response to adjuvant chemotherapy (Figures 6C,E). Additionally, those with poor prognostic features and high risk had much better survival benefits from adjuvant chemotherapy (Figures 6D,E). These results suggest that our 10-lncRNA-based classifier could successfully identify patients with glioma who were suitable candidates for adjuvant chemotherapy.

### Identification of the 10-lncRNA-Based Classifier-Associated Biological Pathways

**Figure 7:**
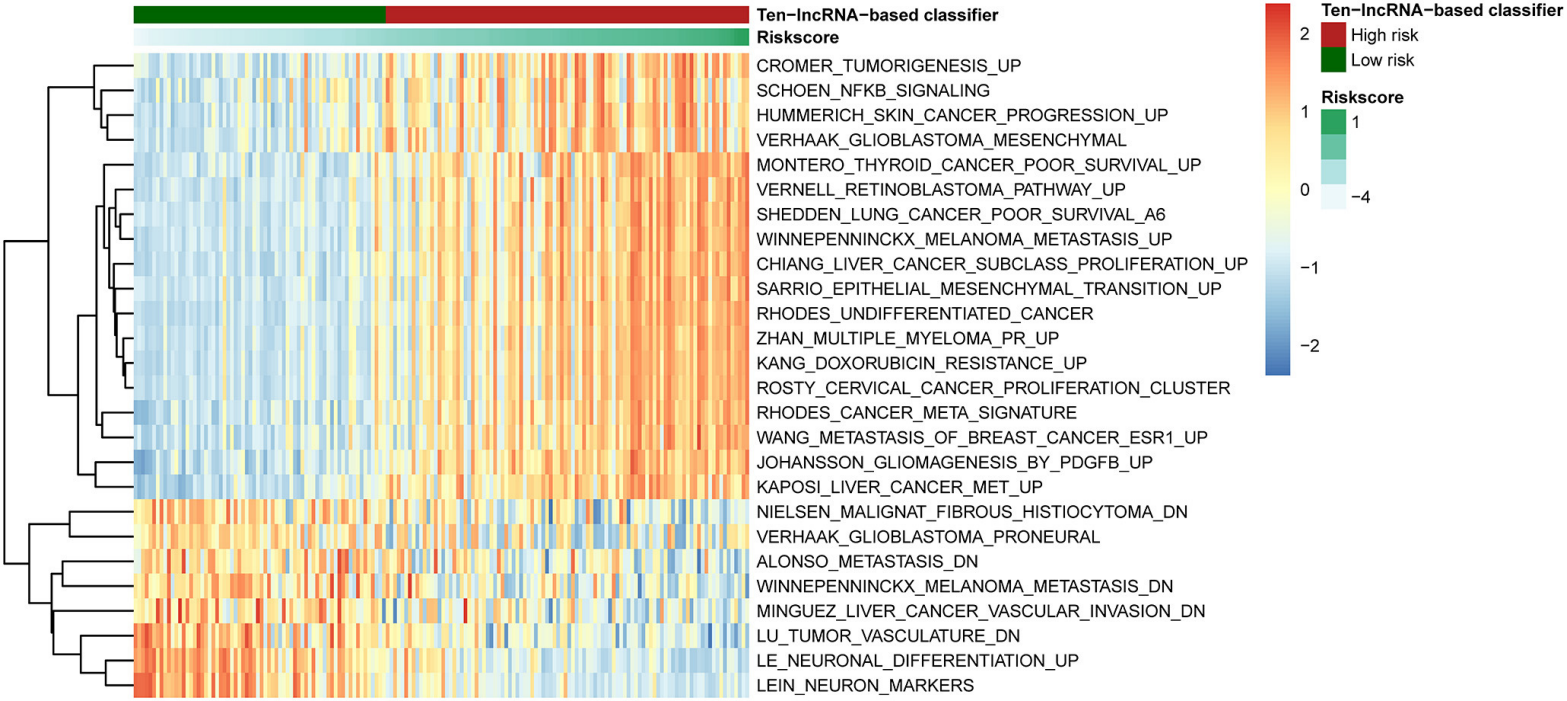
Pathway profile across the training set. Rows represents biological pathways, and columns represent patients with glioma. Each grid represents a score of pathway activity calculated by GSVA. The upper horizontal bar marked the information related to patients, including the risk scores and risk groups.

To detect 10-lncRNA-associated pathways, we used GSVA to search for differentially activated gene sets between high- and low-risk groups. The “GSVA” package was applied to perform the analysis. The results showed that tumorigenesis-, undifferentiated cancer-, epithelial mesenchymal transition-, and poor survival-linked gene groups were enriched in the high-risk group, while patients in the low-risk group were more likely to show less metastasis and tumor vasculature or well-differentiated tumor (Figure 7).

### Clinical Utility of the 10-lncRNA-Based Classifier

**Figure 8:**
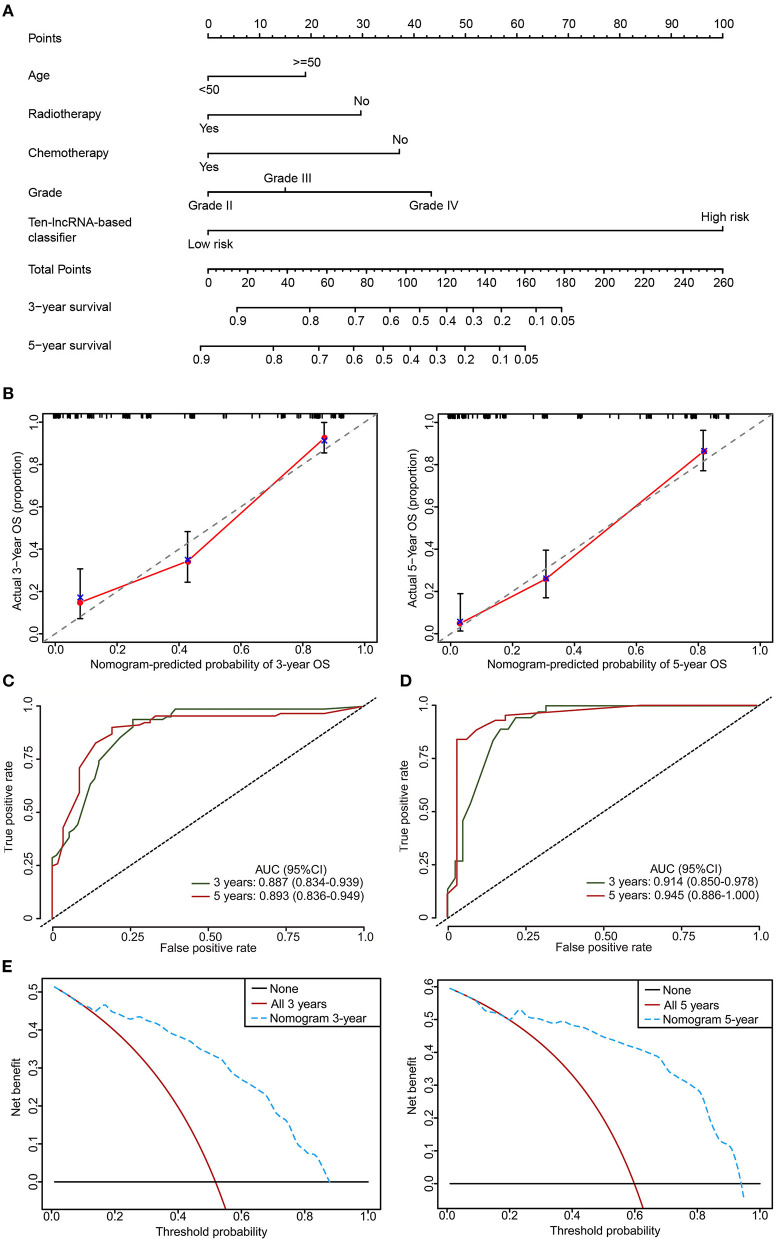
The developed nomogram to predict 3 and 5 year OS probability in glioma. **(A)** The nomogram was constructed in the training set, with the 10-lncRNA-based classifier, age, radiotherapy, chemotherapy, and glioma grade incorporated. **(B)** Calibration curve of the model in terms of agreement between predicted and observed 3 and 5 year outcomes in the training set. Close-ended vertical lines represent 95% CI. The x-axis indicates predicted OS probability, and the y-axis indicates the actual OS. The 45-degree line represents perfect prediction. **(C,D)** Time-dependent ROC curves based on the nomogram for 3 and 5 year OS probability in the training set and the internal testing set. Not all of the clinical factors constituting the nomogram could be obtained in Independent validation sets I and II. **(E)** Decision curve analysis (DCA) for assessment of the clinical utility of the nomogram. The x-axis indicates the percentage of threshold probability, and the y-axis represents the net benefit.

To provide the clinician with a quantitative method for predicting the probability of 3- and 5 year OS in glioma, a nomogram incorporating the 10-lncRNA classifier and four clinicopathological risk features was constructed (Figure 8A). Calibration was applied to check whether the actual nomogram results were close to the expected ones. In Figure 8B, the x-axis denotes predicted survival probability from the nomogram, and the y-axis represents the actual freedom from OS for the patients. The 45° line represents perfect nomogram performance, which represents an outcome likelihood consistent with the actual outcome. Marks plot near the 45° line in a well-calibrated model. The calibration plots showed that the nomogram performed well in the training set and internal testing set when compared with an ideal model (Figure 8B, Figure S6). The predictive accuracy of the nomogram is shown in Figure 7. The AUCs at 3 and 5 years were 0.887 and 0.893 for the nomogram in the training set, respectively (Figure 8C). The internal testing set was used to test the predictive accuracy of the nomogram, which showed that the AUCs at 3 and 5 years were 0.914 and 0.945, respectively (Figure 8D). The nomogram decision curve analysis is shown in Figure 8E. The curve analysis shows that if the threshold probability of 3 and 5 year OS is > 0.11 and 0.22, respectively, the use of the nomogram could offer a higher net benefit than treating all patients or treating no patients.

## Discussion

Here, we established and validated a new prognostic tool on the basis of the expression profile of 10-lncRNAs to improve the prediction of OS and benefit from adjuvant chemotherapy for glioma patients who had already had surgery. It was evident that the classifier could effectively divide patients into high and low-risk groups with large differences in OS. Moreover, the proposed classifier could accurately predict the survival of glioma patients better than each single lncRNA and clinicopathological risk factor. When stratified by these clinical features, the 10-lncRNA-based classifier remains a robust prognostic model, providing prognostic value that complements clinicopathological variables. Furthermore, we built a nomogram for glioma for the first time based on the multi-lncRNA profile and clinical features, which achieved more accurate prediction than clinicopathological features or the classifier alone for 3 and 5 year OS.

One of the advantages of the 10-lncRNA-based classifier over other classifiers is the identification of glioma patients who could benefit from adjuvant chemotherapy. Previous studies indicated that some patients with glioma were resistant to chemotherapy (29, 30). Thus, the key point is to identify patients at high risk who are most likely to benefit from adjuvant chemotherapy. However, we showed that chemotherapy could not improve survival in the clinical high-risk group (glioma patients with any poor prognostic features). We found that adjuvant chemotherapy provides a survival benefit to patients who were classified into the high-risk group by the classifier. Using the classifier might allow for better identification of patients with glioma who are most likely to benefit from chemotherapy. Thus, this classifier provides predictive and prognostic value because patients in the high-risk group have both a worse OS and a clear benefit from adjuvant chemotherapy.

Another advantage of the 10-lncRNA-based classifier is that the 10-lncRNAs were screened from high-dimension gene expression data via LASSO Cox, which results in a robust prognosis value and low correlation among data to prevent overfitting. However, many signatures or classifiers did not use the dimensionality reduction tools to select the genes, which could lead to overfitting (31, 32).

The classification of gene expression data is challenging because the number of genes is large relative to the number of samples. In general, a large number of genes are either irrelevant or redundant and therefore cannot provide classification information. Therefore, reducing the number of genes is of great significance for improving the accuracy of classification. Recently, many studies used machine learning to analyze gene expression data or DNA methylation data to further classify glioma more accurately (33–35). Based on the gene expression data of glioma, Abusamra et al. compared the classification efficiency of three different classification methods, namely support vector machine, k-nearest neighbor, and random forest, and eight different feature selection methods, namely information gain, the twoing rule, sum minority, max minority, the Gini index, sum of variances, t-statistics, and one-dimension support vector machine (36). Recently, LASSO has been extensively applied to Cox proportional hazard regression models for survival analysis of high-dimensional gene expression data (17, 18, 37–40). It can also be used for the optimal selection of variables in high-dimensional gene expression data with a robust prognostic value and low correlation among data to avoid overfitting.

Previous studies have identified prognostic lncRNAs or lncRNA signatures for glioblastoma or low-grade gliomas (9, 31, 32, 41–45). However, these studies have been limited by small sample sizes, small numbers of lncRNAs screened, a lack of independent validation, and the use of unstable statistical models. In this study, using a LASSO Cox regression model allowed us to incorporate multiple lncRNAs into one tool for higher prognostic precision. A total of 1,094 glioma samples were included in this study. In addition, one internal testing test and two independent validation sets were used to validate the classifier.

The biological functions of the 10-lncRNAs used in our study have been investigated in previous studies. LINC00645 is significantly upregulated in malignant endometrial cancer compared to normal endometrium (46). LINC00339 could promote gastric cancer progression by increasing DCP1A expression level via inhibiting miR-377-3p (47). LINC00339 also promoted glioma vasculogenic mimicry formation via regulating the miR-539-5p/TWIST1/MMPs axis (48). HOXD-AS2 promoted glioma progression, and the expression levels of HOXD-AS2 were associated with glioma grade and poor prognosis (49). RHPN1-AS1 could promote uveal melanoma progression and serve as a prognostic biomarker (50). FOXD2-AS1 acted as a sponge of miR-185-5p and influenced the PI3K/Akt signaling pathway by regulating HMGA2, thereby promoting glioma progression (51). A previous study revealed that HAR1A was a prognosic biomarker for diffuse gliomas (52). Low expression and deletion of EPB41L4A-AS1 were found in various human cancers and were associated with poor prognosis (53). Therefore, our 10-lncRNA-based classifier could potentially be used as a predictive tool in personalized therapy and might provide therapeutic targets in the clinical management of glioma.

Moreover, based on the classifier and several clinical features, we constructed a nomogram to predict the 3 and 5 year OS of glioma patients. Despite the fact that such a method normally uses traditional prognostic factors, for instance, age and glioma grade, we propose that including our 10-lncRNA-based classifier could reflect the biological heterogeneity of these gliomas. The nomogram could help clinicians in judging the survival time of glioma patients in the future.

The GSVA results were shown in Figure 7. The epithelial–mesenchymal transition process is crucial in the development of gliomas. The mesenchymal (MES) subtype, one of the glioma molecular subtypes, which expresses mesenchymal biomarkers, has the worst prognosis of the subtypes (54). Moreover, the prognosis of undifferentiated tumors is worse than that of well-differentiated tumors. Our previous study showed that angiogenesis is more common in MES subtype glioma comparing to other subtypes (55).

Our study had several limitations. First, this study is retrospective, and our outcomes need further validation via a prospective study in multicenter clinical trials. Second, lncRNAs reannotated in this study cannot represent the whole lncRNA populations involved in glioma pathogenesis. Lastly, the mechanisms of the 10-lncRNAs were not fully revealed in this study, and further investigations on these lncRNAs are needed to provide in-depth information on their roles in glioma.

This study showed that our 10-lncRNA-based classifier is accurate in categorizing glioma patients into low- and high-risk groups, thus providing a prognostic tool that is complementary to the current clinicopathological risk factors. Furthermore, our study showed that the 10-lncRNA-based classifier can be applied in identifying patients who may benefit from adjuvant chemotherapy. A 10-lncRNA-based classifier nomogram might help clinicians in predicting 3 or 5 year OS probability and in making choices on personalized treatment options for glioma patients.

## Data Availability Statement

The expression datasets analyzed in this study are available in the CGGA, TCGA, and GEO public repositories.

## Ethics Statement

All procedures performed in the studies involving human participants were in accordance with the ethical standards of the institutional and national research committee and with the 1964 Helsinki declaration and its later amendments or comparable ethical standards.

## Author Contributions

Y-BP designed the study and wrote the article. Y-BP, YZ, Q-WZ, and AS analyzed and interpreted the data. All authors reviewed and approved the final manuscript.

## Conflict of Interest

The authors declare that the research was conducted in the absence of any commercial or financial relationships that could be construed as a potential conflict of interest.

## Supplementary Material

Figure S1 Determination of the optimum cut-off value for the 10-lncRNA-based classifier. **(A)** The Pearson correlations of expression levels between the 10-lncRNAs in the classifier were generally weak. **(B–D)** The optimum cut-off value for the 10-lncRNA-based risk scores was determined using the X-tile program in the training set. The colors shown in the plot represent the strength of the association at each division. Red represents an inverse association between the risk score and OS and green represents a direct association.

Figure S2 Univariate analysis based on the 10-lncRNA-based classifier and clinical risk factors in the training, internal testing, and two independent validation sets. **(A)** Training set. **(B)** Internal testing set. **(C)** Independent validation set I. **(D)** Independent validation set II. Solid and black squares represent the HR of death. Close-ended horizontal lines represent 95% CI. We calculated *p*-values using Cox regression hazard analysis.

Figure S3 Validation of the 10-lncRNA-based classifier in the entire CGGA dataset. **(A)** Risk score by the 10-lncRNA-based classifier, patient survival status and time, time-dependent ROC curves, and Kaplan-Meier survival in the entire CGGA dataset. **(B,C)** Univariate and multivariate analysis based on the 10-lncRNA-based classifier and clinical risk factors in the entire CGGA dataset.

Figure S4 Kaplan-Meier survival analysis for patients according to the 10-lncRNA-based classifier stratified by clinicopathological risk factors. **(A)** Internal testing set. **(B)** Independent validation set I. **(C)** Independent validation set II.

Figure S5 X-tile plots of the 10 selected lncRNAs in the training set. Coloration of the plot represents the strength of the association at each division, ranging from low (dark, black) to high (bright red or green). Red represents an inverse association between the risk score and OS and green represents a direct association.

Figure S6 Calibration curve of the nomogram to predict OS probability at 3 and 5 years in the internal testing set.

Table S1 Detailed description of the lncRNAs constituting the 10-lncRNA-based classifier.

Table S2 Clinical characteristics of the 1,094 glioma patients involved in the study.
