# Supplementary figures and images for: Prognostic and Predictive Value of a Long Non-coding RNA Signature in Glioma: A lncRNA Expression Analysis
Source: Front Oncol. 2020 Jul 24;10:1057. doi: 10.3389/fonc.2020.01057 (PMC7394186; doi:10.3389/fonc.2020.01057)

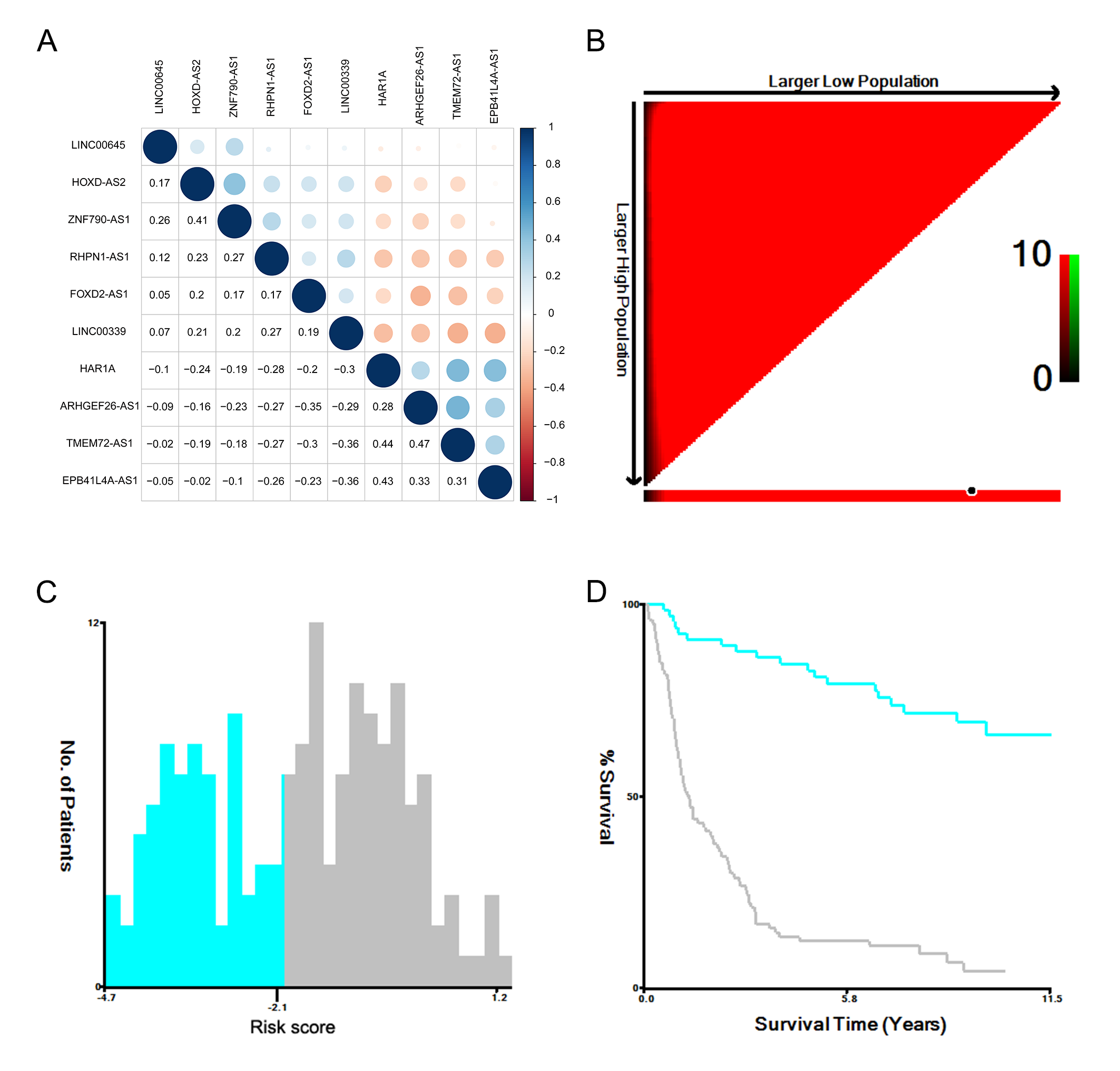

Supplement: Figure S1 — Determination of the optimum cut-off value for the 10-lncRNA-based classifier. (A) The Pearson correlations of expression levels between the 10-lncRNAs in the classifier were generally weak. (B–D) The optimum cut-off value for the 10-lncRNA-based risk scores was determined using the X-tile program in the training set. The colors shown in the plot represent the strength of the association at each division. Red represents an inverse association between the risk score and OS and green represents a direct association. [file Image_1.TIF]

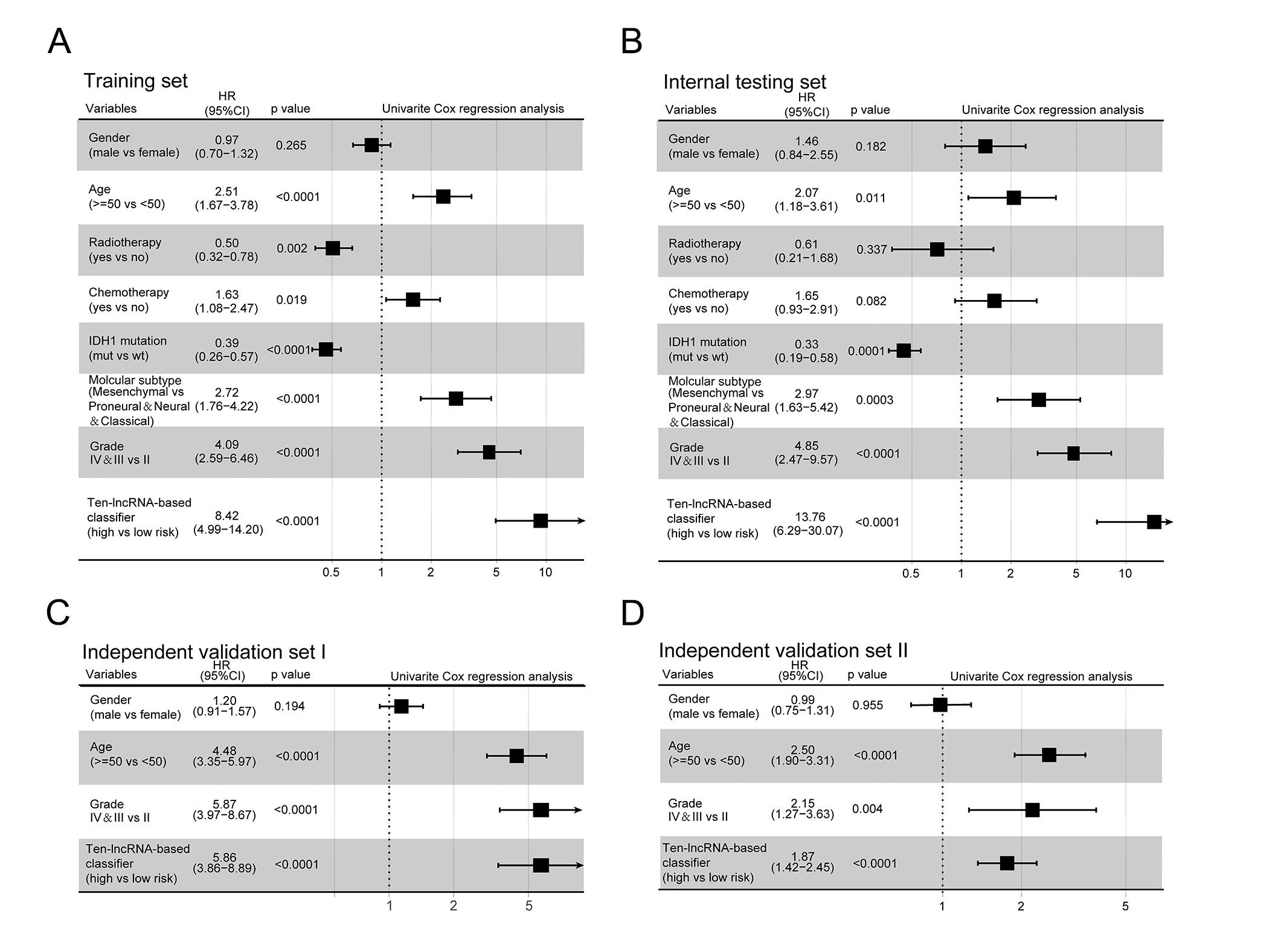

Supplement: Figure S2 — Univariate analysis based on the 10-lncRNA-based classifier and clinical risk factors in the training, internal testing, and two independent validation sets. (A) Training set. (B) Internal testing set. (C) Independent validation set I. (D) Independent validation set II. Solid and black squares represent the HR of death. Close-ended horizontal lines represent 95% CI. We calculated p-values using Cox regression hazard analysis. [file Image_2.TIF]

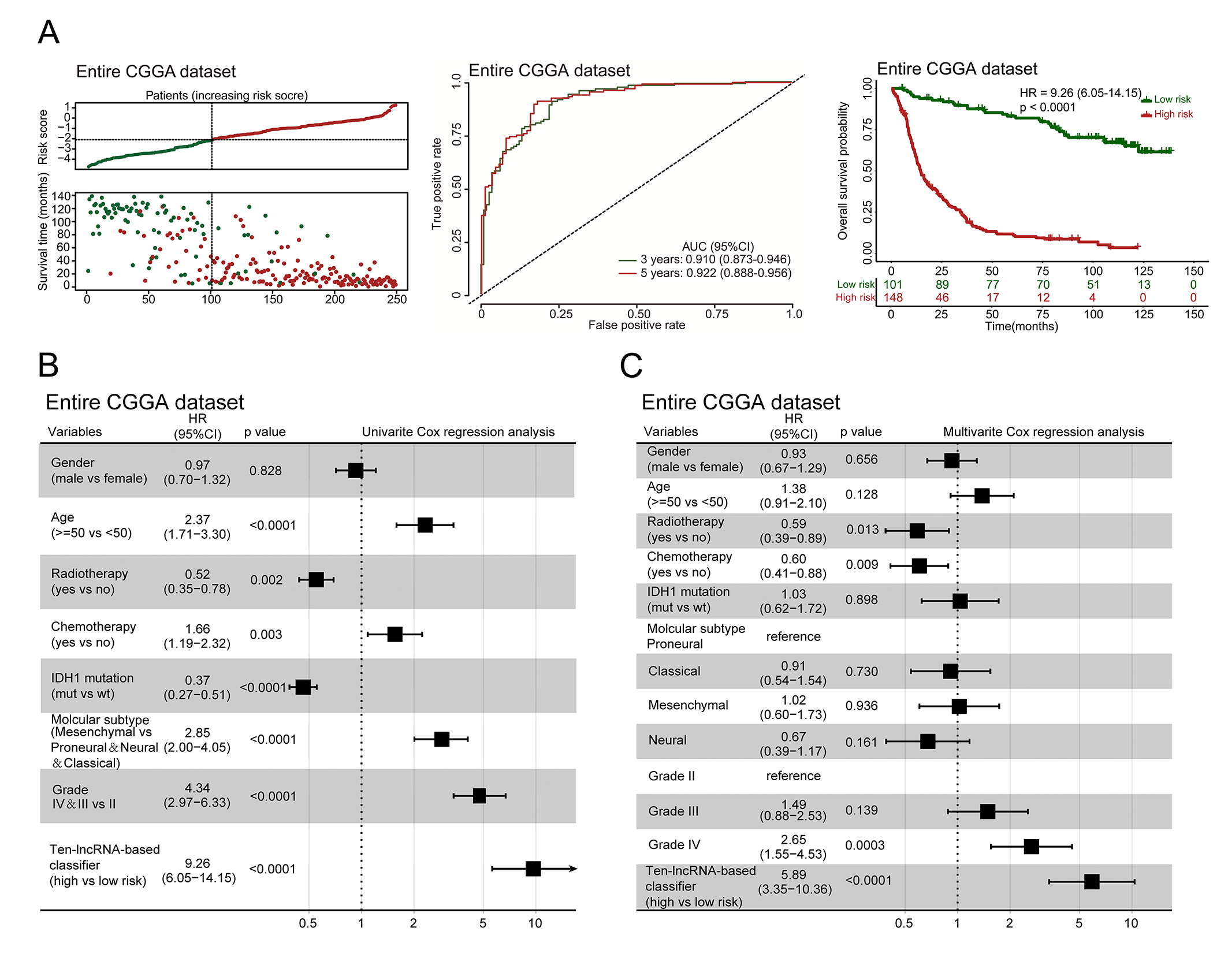

Supplement: Figure S3 — Validation of the 10-lncRNA-based classifier in the entire CGGA dataset. (A) Risk score by the 10-lncRNA-based classifier, patient survival status and time, time-dependent ROC curves, and Kaplan-Meier survival in the entire CGGA dataset. (B,C) Univariate and multivariate analysis based on the 10-lncRNA-based classifier and clinical risk factors in the entire CGGA dataset. [file Image_3.TIF]

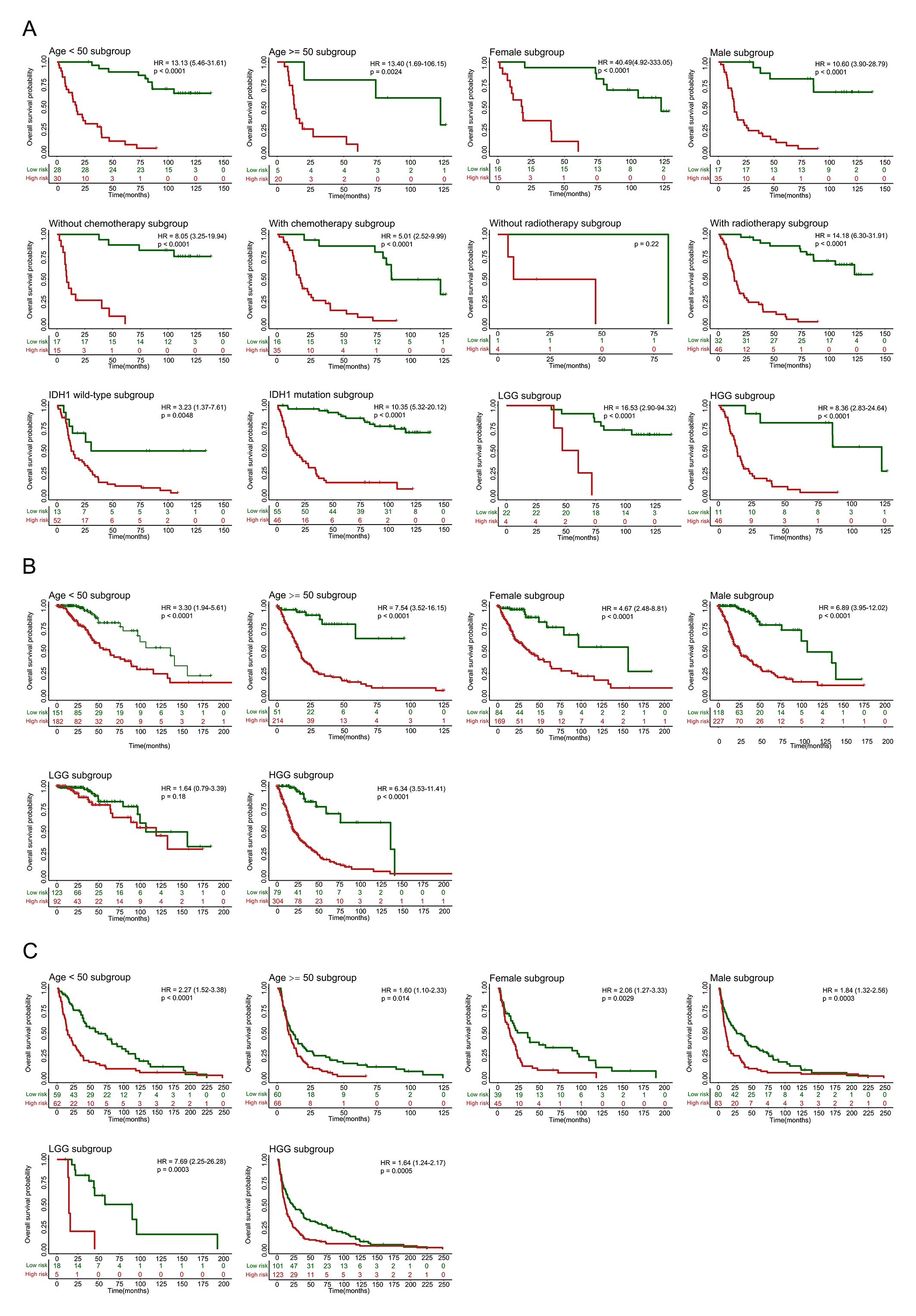

Supplement: Figure S4 — Kaplan-Meier survival analysis for patients according to the 10-lncRNA-based classifier stratified by clinicopathological risk factors. (A) Internal testing set. (B) Independent validation set I. (C) Independent validation set II. [file Image_4.TIF]

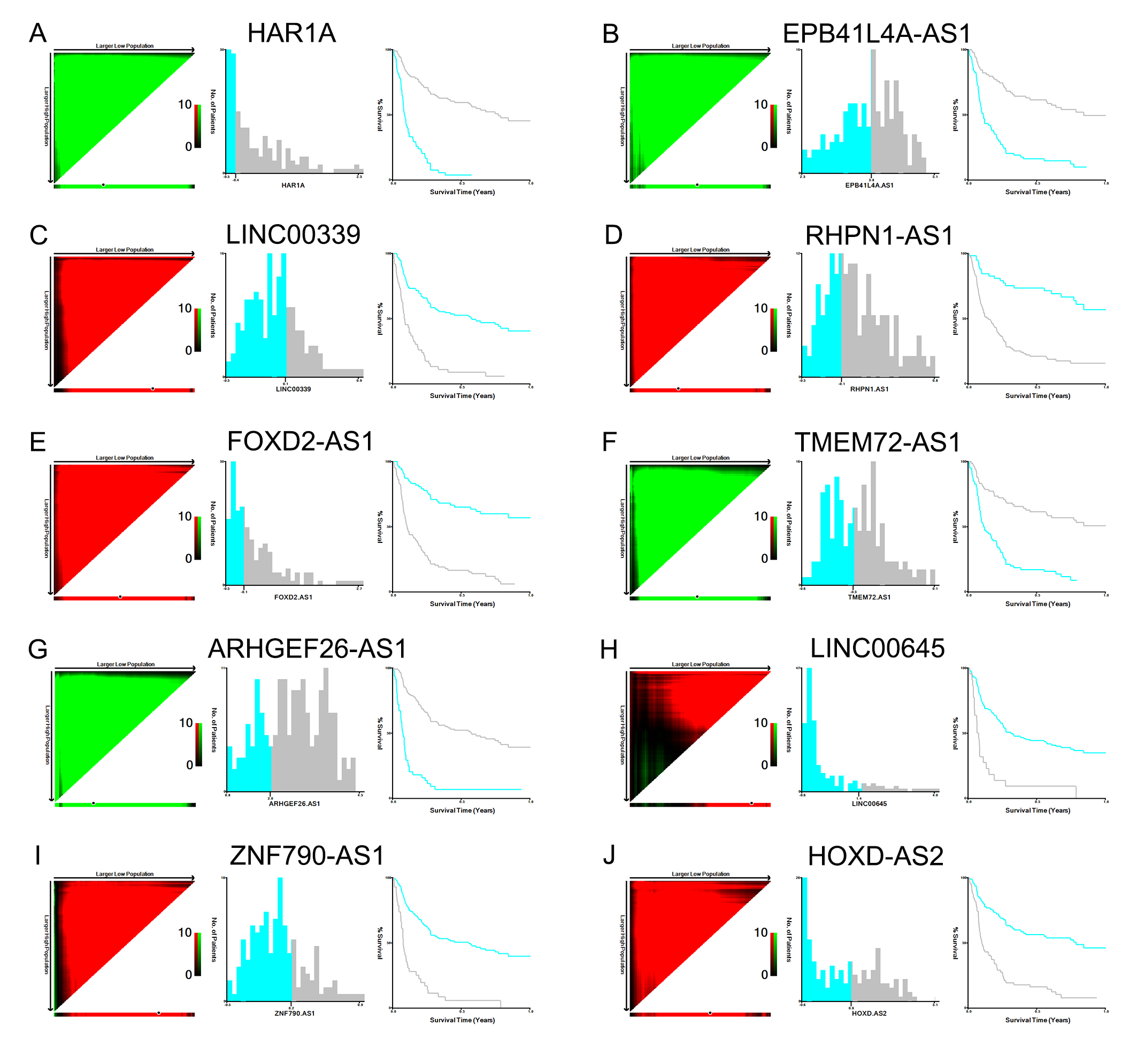

Supplement: Figure S5 — X-tile plots of the 10 selected lncRNAs in the training set. Coloration of the plot represents the strength of the association at each division, ranging from low (dark, black) to high (bright red or green). Red represents an inverse association between the risk score and OS and green represents a direct association. [file Image_5.TIF]

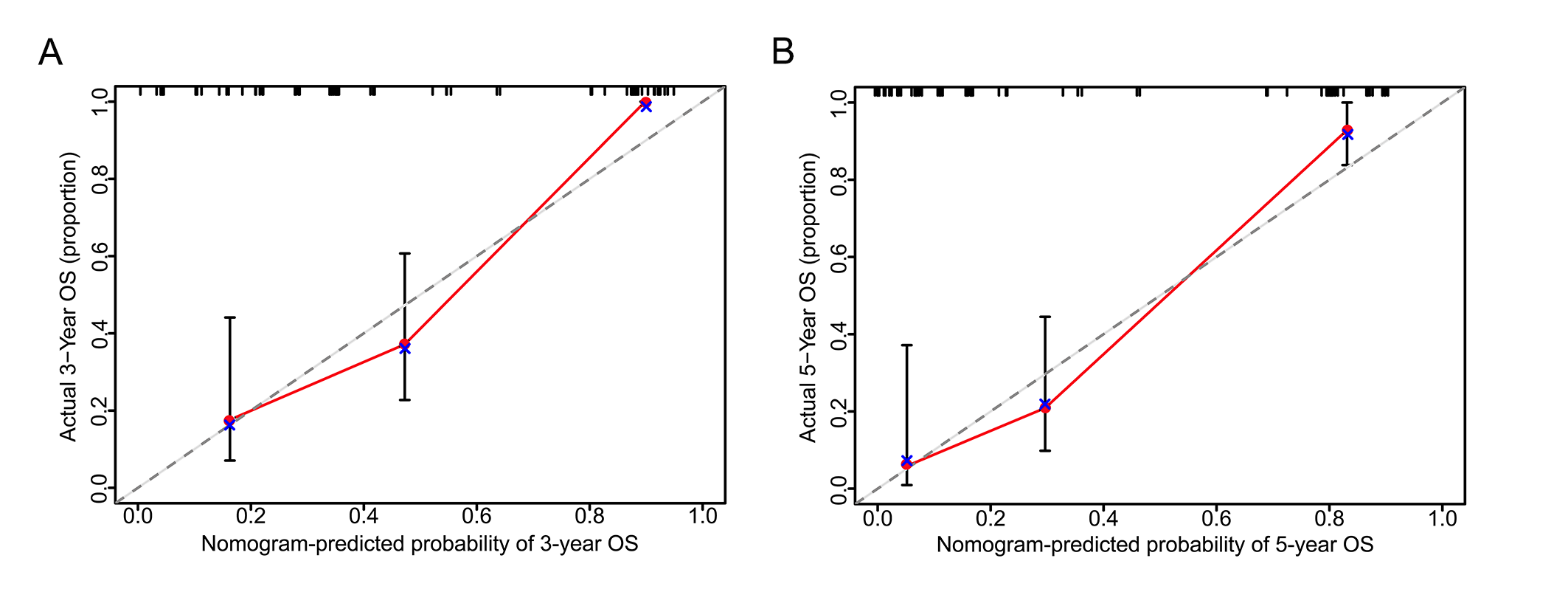

Supplement: Figure S6 — Calibration curve of the nomogram to predict OS probability at 3 and 5 years in the internal testing set. [file Image_6.TIF]
