# Supplementary material for: Prognostic and Predictive Value of a Long Non-coding RNA Signature in Glioma: A lncRNA Expression Analysis
Source: Front Oncol. 2020 Jul 24;10:1057. doi: 10.3389/fonc.2020.01057 (PMC7394186; doi:10.3389/fonc.2020.01057)
Supplement: Table S1 — Detailed description of the lncRNAs constituting the 10-lncRNA-based classifier. [file Table_1.docx]

**Table S1. Detailed description of the lncRNAs constituting the ten-lncRNA-based classifier.**

| **Gene symbol** | **Ensemble ID** | **Description** | **Coefficient** |
| --- | --- | --- | --- |
| LINC00645 | ENSG00000258548 | Long intergenic non-protein coding RNA 645 | 0.353815 |
| LINC00339 | ENSG00000218510 | Long intergenic non-protein coding RNA 339 | 0.800399 |
| ZNF790-AS1 | ENSG00000267254 | ZNF790 antisense RNA 1 | 0.681679 |
| HOXD-AS2 | ENSG00000237380 | HOXD cluster antisense RNA 2 | 0.211079 |
| RHPN1-AS1 | ENSG00000254389 | RHPN1 antisense RNA 1 | 0.120801 |
| FOXD2-AS1 | ENSG00000237424 | FOXD2 adjacent opposite strand RNA 1 | 0.140439 |
| TMEM72-AS1 | ENSG00000224812 | TMEM72 antisense RNA 1 | -1.778034 |
| ARHGEF26-AS1 | ENSG00000243069 | ARHGEF26 antisense RNA 1 | -0.404113 |
| HAR1A | ENSG00000225978 | Highly accelerated region 1A | -0.708967 |
| EPB41L4A-AS1 | ENSG00000224032 | EPB41L4A antisense RNA 1 | -0.413111 |
