# Supplementary material for: Prognostic and Predictive Value of a Long Non-coding RNA Signature in Glioma: A lncRNA Expression Analysis
Source: Front Oncol. 2020 Jul 24;10:1057. doi: 10.3389/fonc.2020.01057 (PMC7394186; doi:10.3389/fonc.2020.01057)
Supplement: Table S2 — Clinical characteristics of the 1,094 glioma patients involved in the study. [file Table_2.docx]

**Table S2. Clinical characteristics of the 1094 glioma patients involved in the study.**

| **Variables** | **Low-risk group (%)** | **High-risk group (%)** | **p-value** |
| --- | --- | --- | --- |
| **Training set (N = 166)** | **N = 68** | **N = 98** |  |
| Age (years) |  |  |  |
| < 50 | 59 (86.8%) | 63 (64.3%) | 0.001 |
| >= 50 | 9 (13.2%) | 35 (35.7%) |  |
| Gender |  |  |  |
| Female | 26 (38.2%) | 36 (36.7%) | 0.844 |
| Male | 42 (61.8%) | 62 (63.3%) |  |
| Radiotherapy |  |  |  |
| No | 11 (16.2%) | 20 (20.4%) | 0.491 |
| Yes | 57 (83.8%) | 78 (79.6%) |  |
| Chemotherapy |  |  |  |
| No | 41 (60.3%) | 25 (25.5%) | < 0.0001 |
| Yes | 27 (39.7%) | 73 (74.5%) |  |
| IDH1 |  |  |  |
| Wild-type | 13 (19.1%) | 52 (53.1%) | < 0.0001 |
| Mutation | 55 (80.9%) | 46 (46.9%) |  |
| Molecular subtype |  |  |  |
| Proneural | 19 (27.9%) | 34 (34.7%) | < 0.0001 |
| Classical | 6 (8.8%) | 27 (27.6%) |  |
| Mesenchymal | 1 (1.5%) | 31 (31.6%) |  |
| Neural | 42 (61.8%) | 6 (6.1%) |  |
| Grade |  |  |  |
| II | 52 (76.5%) | 14 (14.3%) | < 0.0001 |
| III | 13 (19.1%) | 32 (32.7%) |  |
| IV | 3 (4.4%) | 52 (53.1%) |  |
| **Internal testing set (N = 83)** | **N = 33** | **N = 50** |  |
| Age (years) |  |  |  |
| < 50 | 28 (84.8%) | 30 (60.0%) | 0.016 |
| >= 50 | 5 (15.2%) | 20 (40.0%) |  |
| Gender |  |  |  |
| Female | 16 (48.5%) | 15 (30.0%) | 0.088 |
| Male | 17 (51.5%) | 35 (70.0%) |  |
| Radiotherapy |  |  |  |
| No | 1 (3.0%) | 4 (8.0%) | 0.352 |
| Yes | 32 (97.0%) | 46 (92.0%) |  |
| Chemotherapy |  |  |  |
| No | 17 (51.5%) | 15 (30.0%) | 0.049 |
| Yes | 16 (48.5%) | 35 (70.0%) |  |
| IDH1 |  |  |  |
| Wild-type | 5 (15.2%) | 34 (68.0%) | < 0.0001 |
| Mutation | 28 (84.8%) | 16 (32.0%) |  |
| Molecular subtype |  |  |  |
| Proneural | 17 (51.5%) | 14 (28.0%) | < 0.0001 |
| Classical | 0 (0.0%) | 17 (34.0%) |  |
| Mesenchymal | 1 (3.0%) | 16 (32.0%) |  |
| Neural | 15 (45.5%) | 3 (6.0%) |  |
| Grade |  |  |  |
| II | 22 (66.7%) | 4 (8.0%) | < 0.0001 |
| III | 8 (24.2%) | 11 (22.0%) |  |
| IV | 3 (9.1%) | 35 (70.0%) |  |
| **Independent validation set I**  **(N = 598)** | **N = 202** | **N = 396** |  |
| Age (years) |  |  |  |
| < 50 | 151 (74.8%) | 182 (46.0%) | < 0.0001 |
| >= 50 | 51 (25.2%) | 214 (54.0%) |  |
| Gender |  |  |  |
| Female | 84 (41.6%) | 169 (42.7%) | 0.798 |
| Male | 118 (58.4%) | 227 (57.3%) |  |
| Grade |  |  |  |
| II | 123 (60.9%) | 92 (23.2%) | < 0.0001 |
| III | 79 (39.1%) | 159 (40.2%) |  |
| IV | 0 (0.0%) | 145 (36.6%) |  |
| **Independent validation set II**  **(N = 247)** | **N = 119** | **N = 128** |  |
| Age (years) |  |  |  |
| < 50 | 59 (49.6%) | 62 (48.4%) | 0.858 |
| >= 50 | 60 (50.4%) | 66 (51.6%) |  |
| Gender |  |  |  |
| Female | 39 (32.8%) | 45 (35.2%) | 0.593 |
| Male | 80 (67.2%) | 83 (64.8%) |  |
| Grade |  |  |  |
| II | 18 (15.1%) | 5 (3.9%) | < 0.0001 |
| III | 50 (42.0%) | 31 (24.2%) |  |
| IV | 51 (42.9%) | 92 (71.9%) |  |
